# Identification of nutritional composition and antioxidant activities of fruit peels as a potential source of nutraceuticals

**DOI:** 10.3389/fnut.2022.1065698

**Published:** 2023-02-02

**Authors:** Tarique Hussain, Dildar Hussain Kalhoro, Yulong Yin

**Affiliations:** ^1^Institute of Subtropical Agriculture, University of Chinese Academy of Sciences, Changsha, Hunan, China; ^2^Department of Veterinary Microbiology, Faculty of Animal Husbandry and Veterinary Sciences, Sindh Agriculture University, Tando Jam, Sindh, Pakistan

**Keywords:** antioxidant content, sugar content, amino acid content, phytochemicals analysis, mineral content, fruit peel valorization

## Abstract

Fruit peels comprise several biologically active compounds, but their nutritional composition and antioxidant potential of different fruit varieties are limited. This study aimed to determine the nutritional composition and antioxidant properties of 12 peels of different fruit varieties such as apples, pomegranates, guavas, strawberries, grapes, and citrus fruits using a ultraviolet-visible (UV-Vis) spectrophotometer, an inductively-coupled plasma atomic emission spectroscopy (ICP-AES), and an amino acid analyzer. The highest values of TPC, TFC, lycopene, ascorbic acid [total carotenoids and total antioxidant capacity (TAC)], reducing sugars, non-reducing sugars, and total soluble proteins were reported in grapes (Black seedless) 54,501.00 ± 0.82 μM/g dry wt., guava (Gola) 198.19 ± 0.46 Rutin equivalent dry wt., strawberry (Candler) 7.23 ± 0.33 mg/g dry wt., citrus (Mausami) 646.25 ± 0.96 ug/g dry wt., apple (Kala kulu-Pak) 14.19 ± 0.38 mg/g dry wt. and 12.28 ± 0.39 μM/g dry wt., strawberry (Candler) 25.13 ± 0.40 mg/g dry wt., pomegranate (Badana) 9.80 ± 0.43 mg/g dry wt., apple (Kala kullu-Irani) 30.08 ± 0.11 mg/g dry wt., and guava (Gola) 638.18 ± 0.24 mg/g dry wt. compared with its opponent peels of fruits, respectively. All 12 peels of the fruit verities had 20 amino acids and presented as dry matter basis%. The highest trend of glutamic acid + glutamine, glycine, and aspartic acid + asparagine was observed in pomegranate (Badana) 1.20 DM basis%, guava (Surhai and Gola) 1.09 and 1.09 DM basis%, and strawberry (Desi/local and Candler) 1.15 and 1.60 DM basis% in response to other fruit peels, respectively. Regarding the mineral profile, the highest values of nitrogen (764.15 ± 0.86 mg/100 g), phosphorus (53.90 ± 0.14 mg/100 g), potassium (3,443.84 ± 0.82 mg/100 g), ferric (1.44 ± 0.00 mg/100 g), magnesium (1.31 ± 0.00 mg/100 g), and manganese (0.21 ± 0.00 mg/100 g) were found in pomegranate (Badana), grapes (Black seedless), apple (Kala kulu-Pak), and pomegranate (Badana), respectively, in context to other fruit peels’ extract. Principal component analysis (PCA) and agglomerative hierarchical clustering (AHC) were analyzed for determining the correlation among different peels of fruits. Significantly, high levels of variation were noticed among different variables of peels of fruit. Fruit variety and its peels have been distinctive variables in selecting genotypes. The dendrogram obtained from cluster analysis was distributed into two groups and consisted of eight varieties in the same group, and four fruit varieties were in second group. Overall, the results conclude that fruit peels have the abundant antioxidants and some minerals, which can effectively be utilized for nutraceuticals as well as for food security.

## Graphical Abstract

Schematic layout of the experiment. 
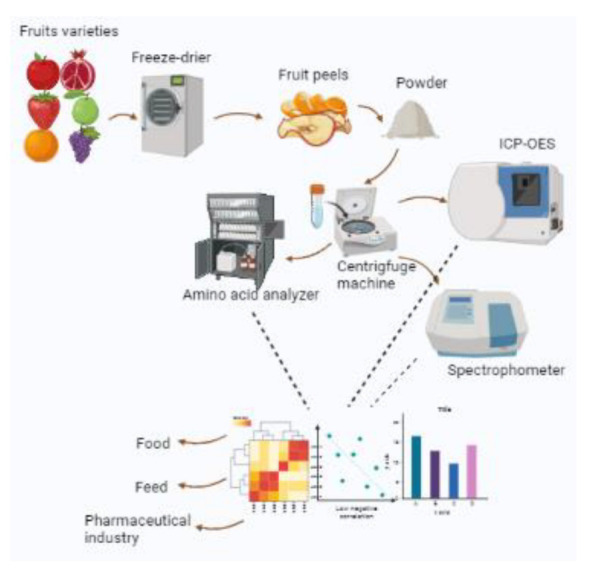

## 1. Introduction

The food industry discards the huge quantities of fruit wastes, particularly peels, seeds, and other fruit leftovers throughout the year (1). Improper disposal of fruit waste and its by-products may create environmental problems and health issues (2). There is an emerging need to properly manage this fruit waste without impacting the environment. Fruit peels have been utilized in products such as agricultural compost, biofuel, and citric acid production (3). Keeping in view that fruit peels are rich sources of carbohydrates, fiber, proteins, and phytochemicals, mainly phenolic compounds (4). The components derived from fruit peels and other fruit parts provide a rich source of valuable antioxidant compounds. Polyphenols, a group of diverse structural compounds mainly present in fruits and vegetables, show significance in human health and nutrition (5). The presence of phenolic compounds exerts high antioxidant potential for the prevention of overwhelming free radicals (6).

Multiple bioactive compounds and their high nutritional value in fruit waste can be further exploited to ensure food security (7). Citrus fruit wastes are rich sources of biologically active substances (8). The citrus fruit peels consist of abundant natural antioxidants such as natural flavonoid, phenolic, ascorbic acid, and carotenoid. The studies report that citrus peel extracts had a greater amount of antioxidant activity than the pulp and seeds (9, 10). The prevalence of bioactive compounds in citrus fruit waste can be utilized as a food additive, prebiotic, pectin source, and dietary fiber. It may also be employed as a natural ingredient for cosmetics, medicines, and several other applications (8). The utmost fruits consumed globally are apples, grapes, and other tropical regional fruits (11). Apple pomace, including peels and others, are a rich source of biological compounds (12). Apples encompass the higher content of flavonoids while the extract from apple has been described the greater antioxidant potential (13). The compounds reported from apple are catechins, procyanidins, phloridzin, phloretin glycosides, caffeic acid, and chlorogenic acid (14). Grapes are the fruits of high nutritional value and bioactive substances (15). Grapes are a potent source of antioxidants consisting of anthocyanin, catechin, epicatechin, resveratrol, and proanthocyanidin, which prevent the formation of free radicals (16). The antioxidant potential of gapes is located in skin and seeds (17, 18). The composition of different compounds including antioxidants may vary according to the grape species, variety, cultivation, climate, and processing factors (19). Pomegranate is a source of bioactive compounds, which has been used since long for medical purposes (20, 21). The pomegranate, particularly the peel, is a rich source of phenols, flavonoids, and antioxidant activities (22). Moreover, the peel has been observed the higher level of antioxidant potential than flower, leaf, and seed (20). At present, pomegranate by-products have attracted huge attention due to having significant amounts of polyphenols (23), which have been reported to have multiple properties *in vivo* and *in vitro* (24). Strawberries have multiple nutritive compounds such as minerals, vitamins, fatty acids, dietary fibers, and other diverse bioactive compounds (19). They have rich sources of ascorbic acid, antioxidant compound, and phenolic such as proanthocyanidins, ellagic acid conjugates, flavonols, and anthocyanins. Moreover, different technologies and processing conditions may affect the phenolic of the fruits (25, 26). The cultivation method and harvesting location can influence the antioxidant potential of the fruit (25). Guava is a fruit, which has been used since long for medical purposes (27). Guava has abundant organic and inorganic compounds such as secondary metabolites such as antioxidants, polyphenols, and others. The high activity of antioxidant has been well-documented (27). Interestingly, these compounds are higher in seeds and skin than the pulp. Existence of these compounds in guava’s food makes it high importance (28). The mechanism of antioxidant activities includes free radical scavengers, quenchers of singlet and triplet oxygen, enzyme inhibitors, and peroxide decomposers and act as synergists (29). Using different extraction and purification tools, it is possible to recover the essential bioactive compounds from fruit waste and converts them into value-added products for industries (30).

Effective utilization of fruit waste or by-products has been used as natural food additives to avoid the disposal of fruit waste and to mitigate environmental problems. More strategies are needed for further exploitation of food additives or supplements with high nutritional value in economical range (31). Scientific innovation in previous decades makes things possible in exploration of more biologically active molecules and their proper utilization of fruit processing by-products. Thousands of molecules derived from fruit waste can be employed in the food, cosmetic, or pharma industry (32). Moreover, keeping in mind that recovery of active ingredients and use of appropriate solvent greatly influence the extraction of functional compounds, therefore, different procedures can be applied for sample extraction. Desirable compounds can be directly utilized as nutraceuticals for their promising health effect or can be employed as raw material for relevant industries. The recovery of diverse compounds from fruit peel waste gives new directions to the industries for their effective utilization of fruit waste for multiple applications. This concept has attracted the attention of developing countries to optimize environment-friendly methods and also a challenge for efficient utilization of these biomaterials to ensure food security (33, 34). This research article aimed to provide the profile of nutritional composition, antioxidants, and biochemical as a potential source of nutraceuticals. According to the best of our knowledge, this is the first descriptive study of local fruits for comparative analysis of nutritional composition and antioxidants from different fruit varieties (peels), and it could be used as a sustainable alternative source for reducing malnutrition in developing countries.

## 2. Materials and methods

### 2.1. Chemical and reagents

The chemicals were utilized in this study for nutritional composition, and antioxidant potentials of different fruit peels were of analytical grade. All chemicals such as acetone, DCIP, Folin–Ciocalteu (FC), gallic acid, potassium phosphate, sodium chloride (NaCl), hydrochloric acid (HCl), nitric acid (HNO_3_), perchloric acid (HCLO_4_), and gallic acid procured from (Sigma-Aldrich NSW, USA).

### 2.2. Sample preparation

In total, 12 different fresh fruit varieties (3–4 kg/variety) that encompass apple (Kala kullu-Irani (KK-I) and Kala kulu, Pakistani (KK-P), pomegranate (Badana and Qandhari), guava (Surhai and Gola), citrus (Kinnow and Mausami), strawberry (desi/local) and Candler), and grapes (Sunderkhani and Black seedless) were procured from local fruit market of Faisalabad, Pakistan. The fruits were manually cleaned, washed twice with distilled water, and dried with tissue papers. The peels of 2–3 kg of each fruit variety were removed, cut into small pieces (0.5 × 1 cm), and then frozen at –20°C overnight, followed by lyophilization at -45°C/50 using the freezer drier (Alpha 2-4 LSC plus–Martin Christ, Germany). The dried extract of the fruit peels was ground to a fine powder and stored at –20°C until further analysis of the following parameters.

### 2.3. Total carotenoids and lycopene

The amounts of total carotenoids and lycopene were measured using the protocol (35). The peels of the fruits (1 g) were homogenized with 10 ml of hexane–acetone mixture (6:4) and incubated at 37°C for 5 min. The contents were then filtered and their absorbance was measured at 453, 505, and 663 nm. The amounts of carotenoids and lycopene were computed using the following equations:


Total⁢carotenoids=0.216⁢A663-0.304⁢A505+0.452⁢A453

Lycopene=-0.0458⁢A663+0.372⁢A505

### 2.4. Ascorbic acid

Vitamin C in the peels of the fruit was estimated using the 2,6-dichlorophenolindophenol (DCPIP) method as described (36). Briefly, each molecule of vitamin C causes the reduction of DCPIP into DCPIPH2 molecule, and this reduction was measured as a decreasing trend in absorbance at 520 nm. A standard curve of ascorbic acid (ASA) was used to measure the concentration in the samples.

### 2.5. Total flavonoid contents and total antioxidant capacity

The total flavonoid (TF) contents were determined as described (37). The peel of each fruit variety in methanol extract (2 mL) was homogenized with 0.1 ml of aluminum chloride (AlCl_3_) (10%), 2.8 ml of deionized water, and 0.1 ml of potassium acetate (1 M). After an incubation of 40 min at 37°C, the absorbance of the assay mixture was measured at 415 nm by a spectrophotometer (UV-VIS U2800, Hitachi, Japan). Rutin was used as the standard, and the TF content was measured as microgram RE gram-1 of the sample.

The TAC of the peel of the fruit extract was standardized using the previous protocol (38). Reagent-1, 1,000 μl of (acetate buffer 0.4 mol/l pH 5.8), take each sample of 25 μl and reagent 2, 100 μl (ABTSS + acetate buffer of 30 mmol/l pH 3.6). After incubation at room temperature for 5 min, absorption of the reaction mixture was read out using a spectrophotometer (660 nm). The first absorbance was taken before the mixing of R1 and R2 (as a blank sample) and the last one at end of the incubation time 5 min after the mixing (39). The ascorbic acid was used as a standard antioxidant.

### 2.6. Total polyphenol contents

The total phenolic content was estimated through a micro-colorimetric method (40), with the use of the Folin–Ciocalteu (FC) reagent. Then, 150 μl of the samples was mixed with 10% FC reagent in addition to 1.2 ml of sodium carbonate. Then, 1 h of incubation at room temperature reading was taken at 765 nm. The linear regression equation was measured using a standard curve, obtained using gallic acid’s fractional concentrations. The total phenolic content (equivalents to gallic acid) of samples was estimated through a linear regression equation.

### 2.7. Total soluble proteins

For protein measurement, each fruit extract was homogenized in 50 mM potassium phosphate buffer (pH 7.0). The protein quantification was done with an earlier method (41). For that purpose, each supernatant of fruit extract of 5 μl and 0.1 N NaCl was mixed with 1.0 ml of Bradford dye and allowed the mixture to stand for 5 min to form a protein-dye complex. Absorbance was calculated at 595 nm with a spectrophotometer. Bovine serum albumin was used as standard.

### 2.8. Measurement of reducing and non-reducing sugars

For the estimation of reducing sugars from fruit peels, the dinitrosalicylic acid (DNS) method (42) was used. The assay mixture was composed of 200 μl of sample extract, 1 ml of DNS reagent, and 1.8 ml of distilled water. After adding the above-mentioned reagents with the sample extract, the reaction mixture was heated in a water bath for 15 min at 100°C, then, the boiled reaction mixture was allowed to cool down at room temperature, and 9 ml of distilled water was added in each test tube. The absorbance of the reaction mixture was finally measured at 540 nm using a spectrophotometer. DNS reagent used for the assay was prepared by adding 96 mM DNS (1 g DNS in 50 ml of distilled water), 30 g of sodium potassium tartrate, 20 ml of 2 N NaOH, and the final volume was made to 100 ml using distilled water. Non-reducing sugars were estimated by the difference in total soluble sugars and reducing sugars.

### 2.9. Amino acid analysis

For the determination of amino acid analysis, each fruit peel extract variety was hydrolyzed using the method (43). The fruit peel extract of each variety (2 or 5 g) was taken in a glass ampoule, 5 ml HCl (6 N) was added to it, and the contents were placed in a hot air oven at 110°C for 24 h. Then, nitrogen gas was passed for the elimination of oxygen from the ampoule to prevent oxygenation. An amino acid analysis was done by ion-exchange chromatography with the help of an amino acid analyzer (Biochrom 30+; Biochrome Ltd, Cambridge, England). Sample hydrolysates were filtered, dissolved in a loading buffer, and then loaded in the amino acid analyzer. The required working conditions of the amino acid analyzer were adjusted according to the manufacturer’s instruction manual. The amino acid analyzer gave the results in the form of peaks. Amino acids were detected by the ninhydrin reaction, identified by their wavelength and retention time, and expressed as a percentage of dry matter.

### 2.10. Mineral profile measurements

Different fruit peels were washed with distilled water and then with deionized water and dried at 65°C in an oven for 48 h and were ground into powder. For the determination of magnesium (Mg), manganese (Mn), and iron (Fe), dried powder of fruit peels was digested in the mixture of nitric acid (HNO_3_) and perchloric acid (HCLO_4_) according to the procedure of Association of Official Agricultural Chemists (AOAC) (44). For the digestion process, 1.0 g of each fruit peel sample was primarily digested in 5 ml of HNO3 (conc.) at the temperature of 100°C on a hotplate until the dark brown fumes had disappeared. After cooling of the mixture, HCLO_4_ (1 ml) was added to each digestion flask and again heated at 180°C until the appearance of dense white fumes of HCLO_4_. The digested mixture was allowed to cool and filter through the Whatman filter paper No.42 and diluted to 50 ml volume using double deionized water. Following digestion, the concentration of elements was determined by direct injection into inductively-coupled plasma optical emission spectrometry (ICP-OES) (PerkinElmer). ICP multi-element standard solution IV, Merck was used for the preparation of a working standard. The fruit peel samples were analyzed for total nitrogen by a standard Kjeldahl method (45). The digested solution was used for the determination of potassium (Flame photometer Jenway PFP7) and phosphorus concentration (46), following the vanadate–molybdate method using a UV-visible spectrophotometer (Specord 210 Plus Analytikjena Germany).

### 2.11. Statistical analysis

All analyses were performed in triplicate. A one-way analysis of variance (ANOVA) using SPSS 20.0 software (SPSS Inc., Chicago, IL, USA) was used, and the differences among treatments were evaluated using Tukey’s test. The results are presented as mean ± standard deviation. Probability values <0.05 were taken to indicate the statistical significance. Moreover, principal component analysis, agglomerative hierarchical clustering, and correlation coefficients were estimated using the computer software Microsoft Excel along with XLSTAT version 2012.1.02., copyright Addinsoft 1995–20121.

## 3. Results

### 3.1. Non-enzymatic antioxidants

The results of non-enzymatic antioxidants and biochemical parameters of fruit peel varieties are displayed in Tables 1, 2. The highest trend of TPC (54,501.00 ± 0.82 uM/g dry wt.) and TFC (198.19 ± 0.46) (Rutin equivalent, μg/g dry wt.) compounds were observed in black seedless grapes and guava (Gola) while the lowest levels were noticed in apple (Kala kullu-Irani), 4,260.23 ± 0.87 uM/g dry wt. and in citrus (Mausami) 177.25 ± 0.32 (Rutin equivalent, μg/g dry wt.), respectively. The maximum values of lycopene were reported in strawberry (Candler) 7.23 ± 0.33 (mg/g dry wt.) and ascorbic acid [646.25 ± 0.96 (ug/g dry wt.)] in citrus (Mausami) while the lowest amounts were found in pomegranate (Qandhari) 1.75 ± 0.48 (mg/g dry wt.) and in strawberry (Desi/local) 604.00 ± 0.82 (ug/g dry wt.) fruit varieties, respectively. The total carotenoids and TAC were reported highest at 14.19 ± 0.38 (mg/g dry wt.) in apple (Kala kulu-Pak) and 12.28 ± 0.39 (μM/g dry wt.) in citrus (Mausami) while the lowest concentration were showed at 6.06 ± 0.12 in guava (Surhai) and citrus (Kinnow) at 6.95 ± 0.62 (μM/g dry wt.) than other fruit peel varieties, respectively.

**TABLE 1:** The non-enzymatic antioxidant status of different fruit peel varieties.

Items	TPC (μ M/g dry wt.)	TFC (Rutin equivalent) (μ g/g dry wt.)	Lycopene (mg/g dry wt.)	Ascorbic acid (μg/g dry wt.)	Total carotenoids (mg/g dry wt.)	TAC (μM/g dry wt.)
Apple (Kala kulu-Pak)	48600.13 ± 0.15^g^	196.61 ± 0.61^d^	7.18 ± 0.25^a^	608.75 ± 0.50^gf^	14.19 ± 0.38^a^	11.56 ± 0.53^ab^
Apple (Kala kullu-Irani)	4260.23 ± 0.87^k^	192.86 ± 0.65^d^	3.32 ± 0.46^cd^	634.0 ± 0.82^b^	7.08 ± 0.11^e^	9.07 ± 0.10^ef^
Pomegranate (Qandhari)	50400.65 ± 0.47^e^	197.03 ± 0.61^ab^	1.75 ± 0.48^ed^	626.0 ± 0.2^d^	6.05 ± 0.11^f^	8.59 ± 0.43^f^
Pomegranate (Badana)	51400.18 ± 0.24^c^	195.25 ± 0.33^c^	6.08 ± 0.10^b^	631.75 ± 0.96^c^	10.08 ± 0.10^c^	8.96 ± 0.06^ef^
Citrus (Mausami)	46500.45 ± 0.33^i^	177.25 ± 0.32^g^	2.52 ± 0.41^de^	646.25 ± 0.96^a^	6.35 ± 0.42^ef^	12.28 ± 0.39^a^
Citrus (Kinnow)	45400.63 ± 0.67^j^	197.28 ± 0.78^ab^	6.27 ± 0.32^b^	608.25 ± 0.96^gf^	12.21 ± 0.25^b^	6.95 ± 0.62^g^
Strawberry (Candler)	51000.55 ± 0.53^d^	196.74 ± 0.53^b^	7.23 ± 0.33^a^	607.00 ± 0.82^i^	12.27 ± 0.50^b^	12.14 ± 0.29^a^
Strawberry (Desi/local)	53800.75 ± 0.50^b^	193.75 ± 0.44^d^	4.04 ± 0.06^c^	604.00 ± 0.82^j^	9.47 ± 0.55^c^	9.66 ± 0.45^de^
Guava (Gola)	53800.50 ± 0.58^b^	198.19 ± 0.46^a^	2.30 ± 0.36^e^	613.75 ± 0.96^e^	8.37 ± 0.44^d^	9.23 ± 0.42^ef^
Guava (Surhai)	49200.75 ± 0.50^f^	189.34 ± 0.47^e^	2.28 ± 0.32^e^	610.25 ± 0.96^f^	6.06 ± 0.12^f^	11.27 ± 0.31^bc^
Grapes (Black seedless)	54501.00 ± 0.82^a^	187.03 ± 0.12^f^	4.05 ± 0.10^c^	633.75 ± 0.96^bc^	14.12 ± 0.14^a^	10.91 ± 0.11^bc^
Grapes (Sundherkhani)	47601.00 ± 0.82^gf^	189.06 ± 0.19^e^	2.25 ± 0.38^e^	633.00 ± 0.82^bc^	13.83 ± 0.23^a^	10.50 ± 0.47^cd^

Values are shown as the mean ± SEM (*n* = 3) and presented in dry weight. Mean values sharing different superscripts which are significantly different.

**TABLE 2:** Biochemical indices of different fruits varieties (peels).

Items	Reducing sugars (mg/g dry wt.)	Non-reducing sugars (mg/g dry wt.)	Total sugars (mg/g dry wt.)	Total soluble proteins (mg/g dry wt.)
Apple (Kala kulu-Pak)	24.80 ± 0.61^a^	3.65 ± 0.24^f^	28.03 ± 0.44^de^	251.33 ± 0.47^k^
Apple (Kala kullu-Irani)	21.85 ± 0.61^bc^	7.63 ± 0.45^b^	30.08 ± 0.11^a^	363.08 ± 0.83^h^
Pomegranate (Qandhari)	25.07 ± 0.49^a^	2.65 ± 0.24^g^	27.91 ± 0.11^de^	294.00 ± 0.01^j^
Pomegranate (Badana)	18.57 ± 0.86^a^	9.80 ± 0.43^a^	29.77 ± 0.21^ab^	523.56 ± 0.54^g^
Citrus (Mausami)	17.23 ± 0.85^a^	9.60 ± 0.22^a^	28.03 ± 0.05^de^	355.34 ± 0.48^i^
Citrus (Kinnow)	22.53 ± 0.60^b^	6.13 ± 0.30^cd^	27.33 ± 0.40^ef^	622.50 ± 0.58^b^
Strawberry (Candler)	25.13 ± 0.40^a^	3.18 ± 0.34^fg^	28.57 ± 0.47^cd^	354.83 ± 0.99^i^
Strawberry (Desi/local)	20.59 ± 0.94^c^	5.83 ± 0.62^de^	27.61 ± 0.43^ef^	552.01 ± 0.73^f^
Guava (Gola)	21.48 ± 0.99^bc^	6.08 ± 0.38^cd^	28.60 ± 0.49^cd^	638.18 ± 0.24^a^
Guava (Surhai)	20.92 ± 0.92^bc^	5.13 ± 0.29^e^	26.96 ± 0.67^f^	617.97 ± 0.07^c^
Grapes (Black seedless)	20.85 ± 0.75^bc^	6.75 ± 0.48^bc^	29.00 ± 0.10^bc^	564.14 ± 0.36^e^
Grapes (Sundherkhani)	22.33 ± 0.69^bc^	3.30 ± 0.30^fg^	26.70 ± 0.48^f^	596.05 ± 0.76^d^

Values are shown as the mean ± SEM (*n* = 3) and presented in dry weight. Mean values sharing different superscripts which are significantly different.

The highest trend of reducing and non-reducing sugar was found in Pomegranate (Qandhari) at (25.13 ± 0.40 mg/g dry wt.) and at 9.80 ± 0.43 (mg/g dry wt.) in Pomegranate (Badana), however, the lowest level was reported in Citrus (Mausami) at 17.23 ± 0.85 (mg/g dry wt.) and at 2.65 ± 0.24 (mg/g dry wt.) in Pomegranate (Qandhari) than other fruit varieties respectively. The total sugars and total soluble protein (TSP) had the highest amounts in apple (Kala kullu-Irani) at 30.08 ± 0.11 (mg/g dry wt.) and in guava (Gola) at 638.18 ± 0.24 (mg/g dry wt.) while the lowest levels were observed at 26.70 ± 0.48 (mg/g dry wt.) in grapes (Sundherkhani) and 251.33 ± 0.47 (mg/g dry wt.) in apple (Kala kulu-Pak) than other fruit peels, respectively.

### 3.2. Amino acid composition

The amino acid composition of different fruit peel varieties is presented on a dry matter basis (%) in Table 3. The highest value of amino acids and leucine was reported at 0.41 and aspartic acid + asparagine at 0.43 on a dry matter basis (%) in KK-Pak and KK-Irani apple varieties. In pomegranate samples, peak values of arginine (0.73) were noticed in Pomegranate (Badana) while the Qandhari variety showed the greatest level of glutamic acid + glutamine [0.88 DM basis (%)], respectively. In Guava varieties, the highest values of glycine were observed (1.09 and 1.33 DM basis%) in Surhai and Gola samples, respectively. The peak concentration of Asp + Asn was showed in Kinnow samples at 0.84 dry matter basis of (%) while this same amino acid was prevalent at 0.71 dry matter basis% in the Mausami variety of citrus fruit peels. In Table 3, proline peak values of grapes (Sundherkhani and seedless) were found (0.17 and 0.12 dry matter basis%), respectively. Aspartic acid + asparagine amino acid was found highest in local as well as Candler varieties of strawberry (1.15 and 1.60 dry matter basis%) than the other fruit samples, respectively.

**TABLE 3:** The amino acid profile of different fruit peel varieties [dry matter basis (%)].

Items	Apple (Kala kulu-Pak)	Apple (Kala kullu-Irani)	Pomegranate (B)	Pomegranate (Q)	Guava (S)	Guava (G)	Citrus (K)	Citrus (M)	Grapes (S)	Grapes (BSL)	Strawberry (D/L)	Strawberry (C)
Cysteine	0.01	0.04	0.09	0.07	0.12	0.26	0.08	0.07	0.03	0.01	0.05	0.06
Methionine	0.03	0.01	0.27	0.20	0.21	0.44	0.10	0.08	0.02	0.01	0.02	0.03
Aspartic acid + Asparagine	0.27	0.43	0.72	0.53	0.49	0.59	0.84	0.71	0.03	0.02	1.15	1.60
Threonine	0.10	0.04	0.19	0.14	0.36	0.44	0.27	0.22	0.03	0.02	0.15	0.21
Serine	0.11	0.12	0.27	0.20	0.42	0.51	0.34	0.29	0.04	0.02	0.19	0.27
Glutamic acid + Glutamine	0.18	0.18	1.20	0.88	0.54	0.66	0.68	0.57	0.08	0.06	0.76	1.05
Glycine	0.12	0.07	1.10	0.81	1.09	1.33	0.35	0.29	0.01	0.01	0.20	0.28
Alanine	0.11	0.04	0.45	0.33	0.38	0.46^*i*^	0.37	0.31	0.07	0.05	0.25	0.35
Valine	0.23	0.04	0.47	0.35	0.43	0.52	0.36	0.30	0.02	0.01	0.15	0.20
Isoleucine	0.26	0.05	0.31	0.23	0.24	0.29	0.27	0.22	0.01	0.01	0.12	0.17
Leucine	0.41	0.06	0.63	0.47	0.41	0.50	0.46	0.39	0.02	0.01	0.27	0.37
Phenylalanine	0.30	0.04	0.63	0.47	0.15	0.18	0.33	0.28	0.01	0.01	0.15	0.20
Histidine	0.01	0.01	0.67	0.49	0.19	0.23	0.17	0.15	0.02	0.01	0.09	0.13
Lysine	0.11	0.06	0.64	0.47	0.06	0.07	0.28	0.24	0.03	0.02	0.20	0.28
Tyrosine	0.09	0.03	0.18	0.13	0.25	0.30	0.23	0.20	0.01	0.01	0.17	0.23
Arginine	0.10	0.04	0.73	0.54	0.42	0.51	0.40	0.34	0.06	0.04	0.22	0.30
Proline	0.33	0.06	0.28	0.21	0.51	0.62	0.69	0.58	0.17	0.12	0.15	0.21
Ornithine	0.02	0.01	0.07	0.05	0.01	0.02	0.07	0.06	0.02	0.01	0.06	0.09

Values are shown as in dry matter percentage. Pomegranate (B), Pomegranate (Badani); Pomegranate (Q), Pomegranate (Qandhari); Guava (S), Guava (Surhai); Guava (G), Guava (Gola); Citrus (K), Citrus (Kinnow); Citrus (M), Citrus (Mausami); Grapes (S), Grapes (Sunderkhani); Grapes (BSL), Grapes (Black seedless); Strawberry (D/L), Strawberry (Desi/Local); Strawberry (C), Strawberry (Candler).

### 3.3. Mineral profile

The mineral profile from selective fruit peels is shown in Table 4, and the values are found in significant amounts. The peak amounts of nitrogen were found in pomegranate (Badana) at 1,524.31 ± 0.52 mg/100 g, pomegranate (Qandhari) at 1312.92 ± 0.64 mg/100 g, grapes (Sundherkhani) at 764.15 ± 0.86 mg/100 g, and guava (Gola) 655.51 ± 0.84 mg/100 g while the lowest was recorded in citrus (Kinnow) 230.84 ± 0.55 mg/100 g, respectively. Phosphorus levels were observed highest in pomegranate (Badana) at 53.90 ± 0.14 mg/100 g, pomegranate (Qandhari) at 42.99 ± 0.22 mg/100 g, grapes (Sundherkhani) at 42.92 ± 0.20 mg/100 g, and grapes (Black seedless) at 42.04 ± 0.69 mg/100 g whereas the minimum was noticed in apple (Kala kullu-Irani) 29.82 ± 0.36 mg/100 g. The potassium, an important element, was noticed greatest in grapes (Black seedless) 3,443.84 ± 0.82 mg/100 g and grapes (Sundherkhani) 3,313.07 ± 0.79 mg/100 g varieties. The value of calcium ranged from 1,013.17 ± 0.81 to 3,443.84 ± 0.82 mg/100 g in citrus (Kinnow) and grapes (Black seedless) fruit varieties, respectively. The iron values ranged from 1.15 ± 0.01 and 1.44 ± 0.00 mg/100 g in strawberry (Desi/local) and apple (Kala kulu-Pak) within all selective fruit peel varieties. The magnesium concentration ranged from 0.46 ± 0.00 and 1.31 ± 0.00 mg/100 g in strawberry (Desi/local) and pomegranate (Badana) fruits’ peel. Moreover, manganese levels were measured and ranged from 0.17 ± 0.00 and 0.21 ± 0.00 mg/100 g in citrus (Mausami), strawberry (Desi/local), guava (Surhai), grapes (Sundherkhani), and pomegranate (Badana), respectively.

**TABLE 4:** The mineral profile of different fruit peel varieties.

Items	N	P	K	Fe	Mg	Mn
Apple (Kala kulu-Pak)	414.11 ± 0.54^h^	32.48 ± 0.07^e^	3106.69 ± 0.64^e^	1.44 ± 0.00^a^	0.58 ± 0.00^e^	0.21 ± 0.00^a^
Apple (Kala kullu-Irani)	426.68 ± 0.88^g^	29.82 ± 0.36^f^	3105.82 ± 0.68^e^	1.31 ± 0.01^b^	0.51 ± 0.00^f^	0.18 ± 0.00^ab^
Pomegranate (Qandhari)	1312.92 ± 0.64^b^	42.99 ± 0.22^b^	2843.52 ± 0.88^g^	1.25 ± 0.01^bcd^	1.28 ± 0.01^a^	0.20 ± 0.00^ab^
Pomegranate (Badana)	1524.31 ± 0.52^a^	53.90 ± 0.14^a^	2853.80 ± 0.70^f^	1.29 ± 0.00^b^	1.31 ± 0.00^a^	0.21 ± 0.00^a^
Citrus (Mausami)	292.85 ± 0.61^k^	32.99 ± 0.19^e^	1266.77 ± 0.69^j^	1.23 ± 0.00^ed^	0.68 ± 0.00^b^	0.17 ± 0.00^c^
Citrus (Kinnow)	230.84 ± 0.55^l^	31.42 ± 0.47^ef^	1013.17 ± 0.81^k^	1.28 ± 0.01^bc^	0.65 ± 0.00^bc^	0.18 ± 0.00^bc^
Strawberry (Candler)	304.79 ± 0.88^j^	36.78 ± 0.08^dc^	3160.19 ± 0.46^d^	1.22 ± 0.01^ed^	0.54 ± 0.00^ef^	0.18 ± 0.00^c^
Strawberry (Desi/local)	383.02 ± 0.85^i^	37.84 ± 0.32^c^	3262.27 ± 0.77^c^	1.15 ± 0.01^f^	0.46 ± 0.00^g^	0.17 ± 0.00^c^
Guava (Gola)	655.51 ± 0.84^d^	35.21 ± 0.72^d^	1981.46 ± 0.57^i^	1.17 ± 0.01^ef^	0.61 ± 0.02^cd^	0.18 ± 0.00^c^
Guava (Surhai)	447.96 ± 0.61^f^	31.69 ± 0.18^e^	2085.89 ± 0.56^h^	1.20 ± 0.00^ef^	0.56 ± 0.0^ef^	0.17 ± 0.00^c^
Grapes (Black seedless)	458.12 ± 0.56^e^	42.04 ± 0.69^b^	3443.84 ± 0.82^a^	1.25 ± 0.01^bcd^	0.52 ± 0.00^f^	0.18 ± 0.00^c^
Grapes (Sundherkhani)	764.15 ± 0.86^c^	42.92 ± 0.20^b^	3313.07 ± 0.79^b^	1.22 ± 0.00^ed^	0.56 ± 0.00^ef^	0.17 ± 0.00^c^

Values are shown as the mean ± SE (*n* = 3). Mean values sharing different superscripts which are significantly different and are presented in mg/100 g of the sample. N, nitrogen; P, phosphorus; K, potassium; Fe, ferric; Mg, magnesium; Mn, manganese.

### 3.4. Principal component analysis (PCA)

Data were analyzed using the principal component analysis. From 12 principal components (PCs), five *viz*., PC-1, PC-2, PC-3, PC-4, and PC-5 had eigenvalues greater than >1 and contributed to 47.37% of entire accumulative variability among the selected fruit peels (Tables 5, 6). The involvement of PC-1 toward variability was greatest (24.973%), followed by PC-2 (22.393%), PC-3 (15.350%), PC-4 (11.917%), PC-5 (9.833%), PC-6 (6.055%), and PC-7 (5.090%) variabilities, respectively.

**TABLE 5:** Principal component analysis of different fruit peel varieties.

	F1	F2	F3	F4	F5	F6	F7	F8	F9	F10	F11
Eigenvalue	3.996	3.583	2.456	1.907	1.573	0.969	0.814	0.280	0.238	0.148	0.036
Variability (%)	24.973	22.393	15.350	11.917	9.833	6.055	5.090	1.751	1.485	0.926	0.226
Cumulative %	24.973	47.366	62.715	74.632	84.466	90.521	95.611	97.363	98.848	99.774	100.000

**TABLE 6:** Contribution of the variables (%) in different fruit peels.

Parameters	F1	F2	F3	F4	F5
N	10.530	11.274	4.528	0.017	2.379
P	7.318	8.589	8.330	6.169	0.213
K	5.035	0.540	1.329	23.450	2.138
Fe	8.504	0.728	19.676	0.002	0.653
Mg	10.295	11.018	0.398	3.710	2.141
Mn	19.993	0.755	2.841	1.168	0.050
TPC	0.602	0.038	21.056	9.190	0.841
TF	11.115	4.759	4.287	8.303	1.842
Lycopenes	5.785	4.964	3.029	1.046	23.168
Ascorbic acid	0.366	16.023	5.299	3.835	2.565
Total Carotenoids	2.179	6.353	0.088	12.381	11.565
TAC	2.113	0.781	2.958	27.528	4.885
Reducing sugar	6.612	14.760	0.087	0.655	8.034
Non-reducing sugar	1.783	14.421	3.617	0.005	18.819
Total sugar	3.391	4.785	4.396	1.523	7.498
TSP	4.379	0.212	18.080	1.020	13.211

N, nitrogen; P, phosphorus; K, potassium; Fe, ferric; Mg, magnesium; Mn, manganese; TPC, total phenolic contents; TF, total flavonoid; TAC, total antioxidant capacity; TSP, total soluble protein.

**FIGURE 1:**
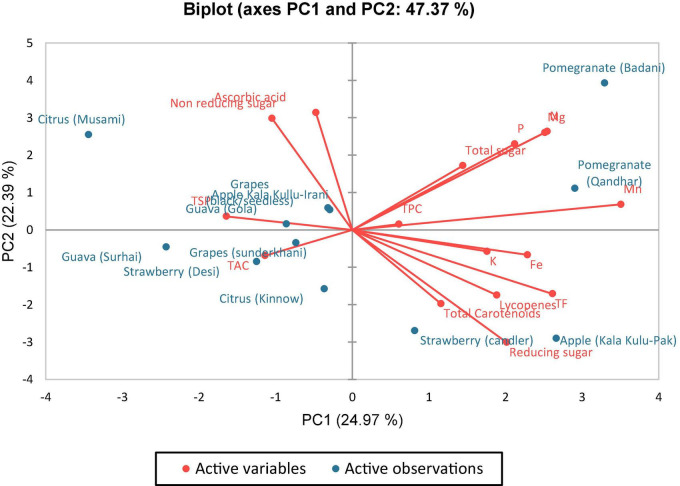
Biplot of different fruit peels compounds for first two principal components.

**FIGURE 2:**
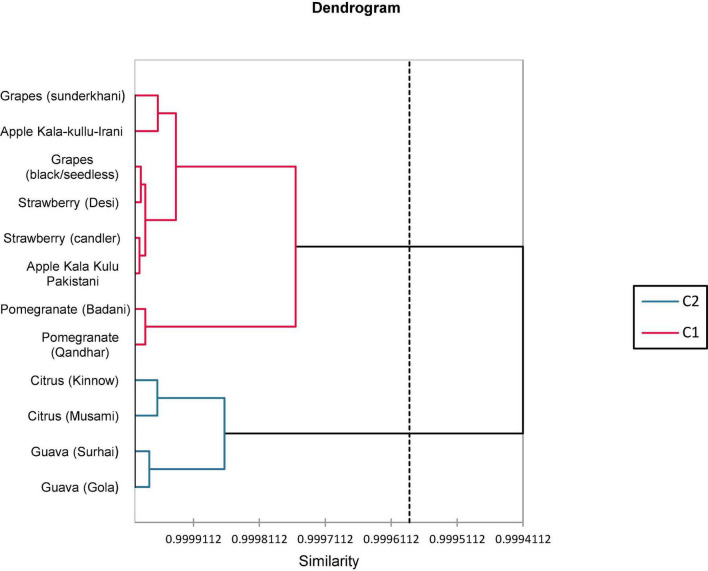
Agglomerative hierarchical clustering (AHC) dendrogram of various fruit peel varieties.

The first and second principal components contributed 47.36% in overall variances, which were plotted on PC-1 (x-axis) and PC-2 (y-axis) to find out the association amid different clusters (Figure 1). It can be viewed that in Figure 2, four plots were made, and there was a positive correlation among all varieties of fruits except guava (Surhai), strawberry (Candler), grapes (Sunderkhani), and citrus (Kinnow), which had negative values compared with other fruit varieties.

### 3.5. Cluster analysis

In total, 12 fruit peels based on the biochemical analysis are presented in Figure 2. Cluster analysis assembled 12 fruit peel varieties into two clusters. Cluster-1 consisted of eight varieties of fruits and cluster-2 comprised four varieties of fruits. In clusters 1 and 2, the similarity and distance of all fruit varieties were mentioned and are shown in Figure 2.

### 3.6. Pearson correlation patterns among different variables

**FIGURE 3:**
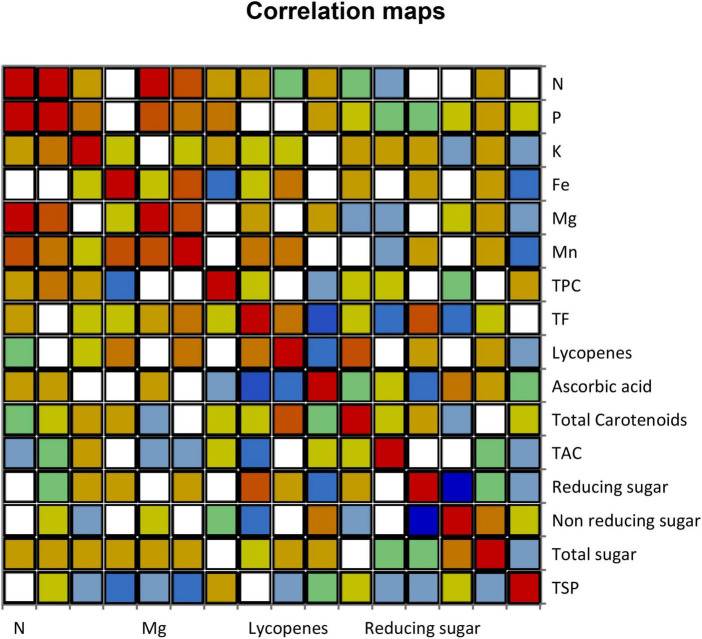
Correlation maps of various fruit peel varieties.

Analysis of variance (ANOVA) showed significant variation among all the parameters of different fruit varieties, and these results found that the variation of different compounds present in fruit peels could successfully be utilized in nutraceutical applications (Figure 3). Nitrogen resulted in a significant positive association with P, Mg, and Mn. Phosphorus had a highly significant correlation with Mg and N, respectively. Potassium showed a significant positive correlation. Ferric revealed a significant positive relationship with Mn. Magnesium observed a significant effect of correlation among P, Mn, and N. Manganese had a significant positive correlation impact toward N, Mg, and Fe. The TPC, ascorbic acid, TAC, non-reducing sugar, total sugar, and TSP indicated a significant positive association of correlations on selective levels. The TF, lycopene, and reducing sugar had a significant positive correlation toward reducing sugar, total carotenoid, and TF, respectively. The coefficient of determination witnessed a significant association among all different variables (Table 7).

**TABLE 7:** Coefficients of determination (Pearson) between biochemical parameters of selective fruit peels.

Variables	N	P	K	Fe	Mg	Mn	TPC	TF	Lycopenes	Ascorbic acid	Total carotenoids	TAC	Reducing sugar	Non-reducing sugar	Total sugar	TSP
N	1															
P	0.728	**1**														
K	0.080	0.178	**1**													
Fe	0.004	0.007	0.015	**1**												
Mg	0.785	0.465	0.002	0.029	**1**											
Mn	0.377	0.160	0.022	0.537	0.536	**1**										
TPC	0.082	0.241	0.115	0.183	0.009	0.001	**1**									
TF	0.060	0.010	0.040	0.038	0.048	0.206	0.035	**1**								
Lycopenes	0.031	0.002	0.013	0.277	0.002	0.170	0.000	0.173	**1**							
Ascorbic acid	0.110	0.095	0.000	0.006	0.088	0.000	0.077	0.455	0.187	**1**						
Total carotenoids	0.023	0.038	0.145	0.130	0.088	0.008	0.033	0.036	0.381	0.038	**1**					
TAC	0.110	0.021	0.053	0.000	0.145	0.057	0.019	0.300	0.001	0.016	0.013	**1**				
Reducing sugar	0.003	0.030	0.121	0.104	0.002	0.090	0.001	0.440	0.080	0.269	0.102	0.006	**1**			
Non-reducing sugar	0.004	0.014	0.127	0.001	0.021	0.002	0.015	0.203	0.000	0.266	0.080	0.010	0.784	**1**		
Total sugar	0.076	0.050	0.075	0.079	0.059	0.113	0.000	0.018	0.058	0.118	0.008	0.011	0.030	0.280	**1**	
TSP	0.001	0.016	0.055	0.320	0.048	0.277	0.086	0.000	0.052	0.029	0.012	0.113	0.145	0.033	0.080	**1**

N, nitrogen; P, phosphorus; K, potassium; Fe, ferric; Mg, magnesium; Mn, manganese; TPC, total phenolic contents; TF, total flavonoid; TAC, total antioxidant capacity; TSP, total soluble protein. Bold values indicate significant differences.

## 4. Discussion

The fruit peel is a barrier to protect the flesh from the environment (47). It avoids dehydration, prevents penetration of pathogens, aids mechanical support, and defends against ultraviolet radiation (48, 49). All these protections are managed by the outer, non-polar layer of the cuticle, and it differs qualitatively and quantitatively within various fruits (50). This study was based on the investigation of antioxidants, amino acids, biochemicals, and minerals from 12 selected varieties of fruit samples. Moreover, the data were subjected to multivariate analysis to find out the correlation within fruit varieties (peels).

The TPC and TFC are bioactive compounds with potent antioxidant potential and could be used as nutraceuticals (51). The TPC and TFC are *in vitro* antioxidant assays; their different values in diverse fruit varieties such as apple, pomegranate, strawberry, guava, citrus, and grapes have been documented, respectively (52–56). TPC is implicated in suppressing oxidant degradation of lipids and preserving the nutritional value of food (51). Our results showed that the highest values of TPC and TFC were reported in black seedless grapes and guava (Gola) while the lowest values were observed in apple (KK-I) and citrus (Mausami) varieties, respectively. These results are in line with the previous results that reported extensive work on phenolic compounds in fruits during storage and ripening periods (57). The same results were documented in gabiroba fruit in Brazil stored at various temperatures (58). Moreover, a reduction in TPC was also been noticed in litchi fruit (59). These variations in concentration occur might be due to several factors consisting of cultivating methods, maturity stages, and methods to be used for analysis.

Lycopene is an antioxidant compound present in plants and fruits (60). Moreover, peels of citrus and grapes are the dominant source of ascorbic acid than their pulp and seeds (61). Table 1 shows the greatest amounts of lycopene and ascorbic acids found in strawberry (Candler) and citrus (Mausami) and more abundantly present than the pomegranate (Qandhari) and strawberry (Desi/local) varieties, respectively. It is suggested that apples (Pak), strawberries (Candler), and citrus (Mausami) are rich sources of lycopene and ascorbic acid. Our results are consistent and in agreement with the previous studies (62). Lycopene is an antioxidant and its health-favored effects have been profound in several diseases (63). Tomato and papaya fruits are rich sources of lycopene (63). Ascorbic acid displays a key role as an antioxidant and it prevents fruit spoilage during ripening due to oxidation. In the previous findings, the highest results of ascorbic acid were noted in pomelo juice followed by grapefruit, lemon, sweet orange, and citron. The difference in variation might be due to ascorbic content in citrus fruits, which never become stable due to tree position, environmental factors, ripening stage, species, and temperature Holcombe (64).

Previous studies have documented that grapes, oranges, guavas, apples, citrus fruits, kiwifruits, and berries are rich sources of total carotenoids, antioxidant activities, etc. (62, 65–67). The different concentrations of total carotenoids and total antioxidant capacity in different fruit peel varieties are illustrated in Table 1. It is advised that apple (Kala kulu-Pak) and citrus (Mausami) had the highest concentration of total carotenoids and total antioxidant capacity. Carotenoids are color-producing agents and possess health-promoting effects. Carotenoids embedded products prevailed in the form of food, feed additive, and supplements. A dietary source of carotenoids improves the animal productivity and health of poultry birds (68). Total antioxidant capacity determines the antioxidant potential of the body. Antioxidants are widely employed for the prevention of diseases. These compounds at molecular level scavenge free radicals and protect the cells from the harmful effects of oxidant products (69). Genetic factors, environmental factors, and physiological phases can modify the composition; concentrations exist in plants, thereby influencing *in vitro* antioxidant properties (70). Previous findings using ABTS assay suggested that white and pink freeze-dried grapefruit peel extracts have strong antioxidant activities and could be used for therapeutic strategies (71). Pal et al. (72) also reported the ABTS radical scavenging capability in kiwi fruit at different ripening stages. Ortega-Arellano et al. (73) also documented ABTS antioxidant potential in Hass and Reed peels, which is similar to the results.

It has been reported that new genotypes improve the nutritional quality of the fruits such as phenolic compounds, vitamins, carotenoids, and other constituents (74). Table 2 indicates the values of reducing and non-reducing sugars, total sugars, and total soluble proteins in all peels of the fruit varieties. Significant variations were observed in all 12 peels of fruit varieties. It is advocated that reducing and non-reducing sugars, total sugars, and total soluble proteins had observed highest in strawberry (Candler), pomegranate (Badana), apple (Kala kullu-Irani), and guava (Gola) while the lowest concentration had noticed in citrus (Mausami), pomegranate (Qandhari), grapes (Sundherkhani), and apple (Kala kulu-Pak), respectively. Most of the fruit varieties have good sources of sugars and total soluble proteins, which could be used for pharmaceutical and nutraceutical purposes. The most common dietary sugars are monosaccharides, galactose, glucose, and fructose, and these all are reducing sugars. Sugars are present in the tissues of most plants. Higher intake of sugar resulted in the onset of obesity, diabetes, cardiovascular disease, and tooth decay. The WHO recommended that sugar must be taken less than 10% in adults and children and minimized below 5% of total energy intake (75). Fruits can have ample amounts of protein but compared with vegetables, beans, nuts, and other high-protein foods, it is less. Moreover, one cup of fruit may provide 1–10% of the daily value for protein. Higher amounts of proteins were reported in guavas, avocados, apricots, kiwifruit, blackberries, oranges, bananas, cantaloupes, raspberries, and peaches (76) while fruit waste could be a good source of proteins to compensate for animal protein requirements (77). Poor-quality proteins also maintain gastrointestinal health and help in the digestion process (77).

Each fruit has its own chemical pattern, such as amino acids, sugars, and organic acids, which contribute to basic cell functions. Secondary metabolites (phenolics, antioxidants, etc.) are usually fruit-specific. These compounds represent nutritional value, aroma, taste, and health-favored effects. They can be potentially employed as indicators for quality, origin, and authenticity of fruit and its derived foods (78). In this study, 18 amino acids both essential and non-essential such as cysteine, methionine, aspartic acid + asparagine, threonine, serine, glutamic acid + glutamine, glycine, alanine, valine, isoleucine, leucine, phenylalanine, histidine, lysine, tyrosine, arginine, proline, and ornithine were found in different peels of fruit varieties ranged from 0.01 to 1.33 on dry matter basis (%). These concentrations were extremely low when compared to the amino acid requirements of the adults and infants (79). The results demonstrated that selected fruit peels are not a sufficient source of amino acids for dietary supplementation. Amino acids are important biomolecules, for maintaining human health. They are known to have promising effects against the diseases such as infertility, intestinal disorder, and neurological dysfunction and could be employed as fingerprints to discover the fruit’s varietal origin (78, 80). Amino acids are aroma precursors in fruit maturation and are used for the synthesis of aroma components (81). It is considered a second key source for the development of volatile aroma compounds (82). Free amino acids are essential for food flavoring, improving its palatability, and helping in the development of amines and volatile compounds (83). For example, two amino acids such as tyrosine and phenylalanine may be the substrates for further improvement in aroma compounds (84). The prevalence of amino acids such as Gly, Ala, and Pro impacts the fruit taste and thus produces a sweet flavor (81).

It has been known that fruit peels are rich sources of minerals. The citrus fruit peels can be employed for mineral preparations of varying compositions and properties. So, the use of minerals has health-benefiting effects (85). Table 4 represents the six minerals such as nitrogen, phosphorus, potassium, iron, magnesium, and manganese measured in 12 different peels of fruit varieties, and the results are shown in mg/100 g. It is suggested that three minerals such as nitrogen, phosphorus, and potassium were found significantly highest in selective fruit peel extracts (86). Potassium elements contribute to the regulation of electrolyte balance and the acid-base balance in the body (87, 88). A previous finding by Barros et al. (89) showed that the peel of orange, lime, and lemon accumulates more potassium than the pulp. The literature revealed that phosphorus, potassium, and nitrogen are the key elements, which significantly affect the characteristics of fruit (90). Unfortunately, none of the literature on mineral profiles was published on the peels of these selective fruit varieties. The iron concentration was reported higher in oranges (86). Liu et al. (91) documented that grapefruit and citrus fruit had the lowest iron. It is observed that 1–5% of the total iron was found in fruits that are available for humans and is largely affected by the prevalence of other food compounds (91). The fruits which reported a greater amount of magnesium are mandarin and orange as revealed (86, 92). The differences in the results might be due to several factors such as the type of soil the fruit was grown and that a significantly greater amount of manganese was found in pomelos in comparison with other fruits and also in oranges and mandarins (85, 90). Likewise, the same findings in which maximum contents in orange, mandarin and lemon were reported. Of note, a minimum level of manganese was detected in lime and over three times lower than in orange and pomelo (86).

The principal component analysis reports the main significant contributors to overall variation in each differentiation axis (Figure 1). The eigenvalues support in describing all the variables from the selected fruits (peels) that can be engaged. The eigenvalues are higher in F-1, F-2, F-3, F-4, and F-5 and are equivalent to the level of variables (93), and these variables strongly correlated with each other. Numerical data having higher absolute values close to the unity in the first PC influence the grouping in comparison with those that had a lower absolute value close to zero (93, 94). In this study, out of 12 PCs, five *viz*. PC-1, PC-2, PC-3, PC-4, and PC-5 had eigenvalues > 1 and contributed 47.37% of total cumulative variability among different peels of fruit varieties. The PC-1 enroute to variability was the highest (24.973%). The PC-2 observed (22.393%), PC-3 (15.350%), PC-4 (11.917%), PC-5 (9.833%), PC-6 (6.055%), and PC-7 (5.090%) variabilities, respectively, for N, P, K, Fe, Mg, Mn, TPC, TF, lycopenes, ascorbic acid, total carotenoids, TAC, reducing sugar, non-reducing sugar, total sugar, and TSP. Hence, once a variable is elected from these clusters rely on individual loadings (94). Hence, Mn is the premium choice that had the greatest contribution to PC-1 while ascorbic acid is the key contributor to PC-2 and Fe in PC-3, respectively. These findings obviously indicated that the peels of the fruit varieties are a good source of antioxidants and biochemical and could be a rich source of nutraceuticals.

A clustering analysis was applied to estimate the degree of similarity among 12 peels of fruit varieties as illustrated in Figure 2 as dendrograms and clustering observation among the peels of fruits. Accordingly, the fruit verities were classified into two main groups: eight varieties of fruits were included in the first group whereas the remaining four varieties were encompassed in the second group. The similarities among the differences between the peels of fruit varieties observed that clustering analysis (CA) disclosed a correlation assessed through PCA in terms of biochemical variables. Pereira-Netto (95) described that tropical fruits had mutual similar greater concentrations of phenolics compared with temperate fruits that admit with the clustering, in which tropical fruits such as mango peel, dragon fruit peel, lime peel, and custard apple peel were fall in the same group.

A total of 47.39% variability of the initial data can be deciphered by the compiling first two factors (F1 and F2) as shown in Table 7. Antioxidant compounds were strongly associated with each other. This significant association was not in line with the findings of Floegel et al. (96) who reported that DPPH and ABTS assays examine the free radical scavenging ability while ABTS may better reflect the hydrophilic, lipophilic, and high amount of antioxidants in fruits in response to DPPH assay. The high association among TAC with phenolic, flavonoid, lycopene, ascorbic acid, and total carotenoid present in 12 different peel extracts of fruits elicit potent scavenging ability, respectively. Our study is the first evidence, which shows a positive correlation among different compounds of peels of fruits, but these results were not in agreement with the previous results.

The N was highly significantly correlated with three minerals such as P, Mg, and Mn. The P had a high significant association with N and Mg. The Fe had a great significant relationship with Mn. The Mg had a strong significant correlation with N, P, and Mn. The Mn had a high significant association with N, Fe, and Mg. TF and total carotenoids were a positive significant correlation with reducing sugar and lycopenes. Reducing sugar had a significant positive association with TF, while K, TPC, TAC, non-reducing sugar, total sugar, and TSP had significant effects, but none of the correlation was found with other varieties of fruits. The results conclude that selective parameters had a highly significant effect and strong correlation with other variables that could be used for nutraceuticals.

## 5. Conclusion

All these peels of fruit varieties had a low profile of amino acids and some minerals but have a strong *in vitro* antioxidant capacity and biochemical indices. The TPC, TFC, lycopene, ascorbic acid, total carotenoid, and TAC had higher biological activities in grapes (Black seedless), guava (Gola), strawberry (Candler), citrus (Mausami), apple (Kala kulu-Pak), and strawberry (Candler) than the other fruit peels, respectively. These results supported the concept that fruit peels are a strong source of food waste, having potent antioxidant properties, and there is a dire need for effective utilization in nutraceutical approaches and feed formulation. Moreover, advanced techniques such as HPLC, NMR, and LC-MS/MS will also be employed for further characterization of phenolic compounds present in these fruit varieties. For commercialization, *in vitro* digestibility, bioavailability, bio-accessibility, nanoparticle-embedded approaches, toxicological, and animal studies are warranted using different fruit peels.

## Data availability statement

The raw data supporting the conclusions of this article will be made available by the authors, without undue reservation.

## Author contributions

TH: conceptualization and drafting. DK: conceptualization, administration, statistical analysis, and editing. YY: editing of the manuscript. All authors evaluated and approved the manuscript for publication.
